# Supplementary material for: Myxobacteria restrain Phytophthora invasion by scavenging thiamine in soybean rhizosphere via outer membrane vesicle-secreted thiaminase I
Source: Nat Commun. 2023 Sep 13;14:5646. doi: 10.1038/s41467-023-41247-0 (PMC10499793; doi:10.1038/s41467-023-41247-0)
Supplement: Supplementary file 1 — Supplementary Information [file 41467_2023_41247_MOESM1_ESM.pdf]

Supplementary Information

Myxobacteria restrain *Phytophthora* invasion by scanvaging thiamine in soybean rhizosphere via outer membrane vesicles-secreted thiaminase I

Supplementary Methods

Isolation and identification of thiamine-producing bacteria

Soybean rhizosphere soil from four different fields were collected for the isolation of microorganisms using the dilution plating method. Briefly, the collected soil samples from soybean fields were vortexed within deionized water for 30 min at 150 rpm. The mixture was left to stand at room temperature for 3 min, and the supernatants were serially diluted in sterile 0.85% NaCl solution, after which the resulting suspensions were spread on Luria-Bertani (LB) agar plates, 1/10 tryptic soy medium, and Reasoner's 2A agar medium at 30 °C for bacterial screening[1]. A total of 1365 isolates were recovered from the soil samples. Thiamine-producing isolates were further screened on CG medium (1% casein hydrolyzate, Vitamins free; 0.2% glucose) containing the thiamine auxotrophic strain K-12 Δ*thiE* (10^6 CFU/plate) as the reference strain. The co-culture was incubated at 30 °C for 2 d. If the isolate could produce thiamine, the strain K-12 Δ*thiE* around the colony of the isolates will resume growth, resulting in the formation of an opaque circle. Thiamine production was classified according to the diameter of the opaque circle, more than 2 cm for + + +, 1 to 2 cm for + +, 0-1cm for +, no circle for -. Single colonies with turbidity at the bottom were picked for liquid culture in CG medium. The culture supernatant of each screened isolate was harvested by centrifugation at 10000 g and passed through a 0.22 μm sterile filter to remove remaining cells. Then, 250 μL of the cell-free supernatant was added to 250 μL of MM[2] medium to determine the effect on the growth of strain K-12 Δ*thiE*. The thiamine-producing bacteria were further identified by 16S rRNA gene sequencing.

Representative strains are shown in Supplementary Table 6.

Collection of soybean root exudates

Soybean root exudates were collected following a slightly modified procedures as described by Liu *et al*[3]. Three different soybean varieties (25 plants each of Hefeng-47, Huning 95-1 and Lvling-9) were selected for the assays. The soybean seeds were surface sterilized with 3% sodium hypochlorite for 10 min and rinsed with sterilized water, after which the seeds were sown in sterilized soil and grown under greenhouse conditions (16 h-light/8h-dark, 80% relative humidity) until full expansion of the first true leaf (10-12 d). Seedlings were washed and transplanted to MS[4] culture at 25 °C for 12 h. To collect the root exudates, the soybean plants were rinsed with sterile water and the roots were placed in 200 mL of sterile water at 25 °C for 12 h in the dark. The collected solutions were filtered through a 0.22 μm filter and lyophilized. The lyophilized contents were re-dissolved in 2 mL of sterile water and the thiamine content was determined using the bioassay.

Preparation of zoospores and the lysates

Phytophthora sojae wild-type P6497 was cultured on 10% V8 agar plates at 25 °C for 7 d, following by repeatedly washing with sterile water and incubating the washed mycelia in the dark at 25 °C for 30 min until sporangia developed on most of the hyphae[5]. The numbers of the zoospores were counted directly via light microscopy. For preparation of the zoospores lysates, the zoospores was suspended in 1 mL H_2O after centrifugation at 2500 g with a concentration of 2×10^7 cells/mL, following by homogenization using the Bioprep-24 Homogenizer (40 s, speed 6.00M/S, 3 times).

The supernatant was obtained after centrifugation and used as the zoospores lysates.

Scanning electron microscopy analysis

To ascertain the effects of strain EGB and SUPL on the cell morphology of P6497 in liquid culture, a scanning electron microscope (SEM, Hitachi SU8010) was used to observe the integrity of the cell wall. The method for preparing the SEM samples was described previously[6]. Briefly, mycelia were fixed with 2.5% (v/v) glutaraldehyde at 4 °C and washed three times with deionized water. Then, the samples were dehydrated by incubation in 70%, 85%, 95% and 100% ethanol, respectively, followed by gold sputtering for SEM analysis.

Biochemical properties

To determine the temperature stability of the active factor, 10 μL SUPL (1 mg/mL) was incubated with 90 μL 10% V8 medium at different temperatures (20-70 °C) and the residual antimicrobial activity against *Phytophthora* was measured using the 96-well growth assay at 25 °C. The pH stability was determined by incubating SUPL (1 mg/mL) in different buffers (sodium citrate buffer, pH 3.0-7.0; Tris-HCl buffer, pH 7.0-9.0; sodium-glycine buffer, pH 9.0-10.0) at 4 °C for 12 h, and adjusted pH to 7.0 with Tris-HCl buffer for 96-well plate assay to measure the residual activity. The protease stability was determined by incubating SUPL (1 mg/mL) in different protease solutions (Protease K; Papain; Trypsin; 1 mg/mL dissolved in 50 mM Tris-HCl buffer pH 7.2) at 30 °C for 3 h, and the residual antimicrobial activity against *Phytophthora* was measured as described above. To determine the distribution of active fractions in different cellular component, the SUP, SUPL, SUPU (OMVs-free SUP) and OMVs

were isolated, and the antimicrobial activity against *Phytophthora* was measured using the 96-well assay at 25 °C.

Antimicrobial assay of CcThi1

To evaluate the effect of CcThi1 on the growth of filamentous fungi, Potato Dextrose Agar (PDA) was melted and cooled to about 40 °C. Then, CcThi1 in Tris-HCl buffer (pH 7.2) was added to the medium to a final concentration of 0.24 μM, shaken well and poured onto the plates. Tris-HCl (pH 7.2, 50 mM) buffer was used as control. After the plates were solidified, 0.2×0.2 cm agar blocks were cut from the growing edge of the colony and inoculated at the center of the PDA plates with 0.24 μM CcThi1. The growth of fungi was observed after incubation at 28 °C for 5-7 d. To test the effects of CcThi1 on the growth of bacteria and yeasts, the indicator strains were grown in Luria-Bertani (LB) and Yeast Peptone Dextrose (YPD) medium at 30 °C for 2 d, after which the cells were obtained by centrifugation and washed 3 times with sterile water, after which 100 μL of a suspension comprising 10^6 cells/mL were spread evenly on top of LB or YPD plates. After the plate surface dried, an Oxford cup was placed at the center of the plate with 200 μL CcThi1 (11.9 μM) or 50 mM Tris-HCl (pH 7.2), followed by incubation at 30 °C for 24 h. For oomycetes, the melted 10% V8 medium or 10% V8 medium with rye sucrose agar (RSA)[7] (for *Phytophthora infestans*) was cooled to 40 °C. Different concentrations of CcThi1 were added into the plates, and 50 mM Tris-HCl buffer (pH 7.2) was used as a control. After solidification, the mycelial block (0.2 × 0.2 cm) was removed from the growing edge of the *Phytophthora* colony and transferred to the center of the plates. The growth of *Phytophthora* was

observed under a stereomicroscope after incubation at 25 °C for 7-12 d, and the colony diameter was measured.

Genetic manipulation of *M. xanthus* DK1622

The in-frame deletion of the *mxthi1* gene (GenBank: ABF86831.1) of *M. xanthus* DK1622 was performed as described previously[8]. Briefly, a 1181-bp upstream fragment and 1119-bp downstream fragment of the target gene were amplified by PCR and cloned into the plasmid pBJ113. Correct plasmids were introduced into DK1622 by electroporation (Electro Cell Manipulator ECM 630; BTX). The electroporation parameters were 1.25 kV, 400 Ω, 25 μF, and 2-mm cuvette gap. The insertion of plasmids after the first homologous recombination was screened on CTT agar supplemented with 40 μg/mL kanamycin, followed by counter-selection on CTT plates with 2% D-galactose (Sigma). Validation of in-frame deletion strains (CL1003) was performed by PCR and western blotting.

For ectopic expression, the full-length *ccthi1* gene from strain EGB and *mxthi1* from strain DK1622 was cloned into the pSWU19 vector with the *pilA* promoter and integrated by site-specific recombination at the *attB* site. The plasmid was introduced into CL1003 using the electroporation conditions described above. The primers and plasmids are listed in Supplementary Tables 3 and 4.

Western blot analysis

The peptide sequence CGGRPYPNQRFPENR derived from CcThi1 was synthesized to produce anti-CcThi1 antibodies by immunizing rabbits (GenScript, China), the peptide of CcThi1 shares 76.66% sequence identity with MxThi1 of

DK1622. The western blot analysis was carried out according to the manufacturer's instructions (Bio-Rad). Briefly, the protein concentration of the samples was determined using a BCA assay kit, and 12% acrylamide SDS-PAGE analysis was performed with a constant total protein content. After electrophoresis, the gel was blotted onto a NC membrane for immunostaining, and blocked with 5% skim milk. The membrane was incubated with the anti-CcThi1 antiserum (0.5 μg/mL) at 1:5000 followed by the secondary anti-rabbit IRDye 800CW antibody (Odyssey, catalog no. 926–32211) at 1:10,000. The membrane was visualized using an Odyssey (LI-COR) scanner with excitation at 700 and 800 nm to detect the specific bands.

UPLC-MS

The reaction solutions (10% V8 medium without nucleophile or SUPL treatment) were lyophilized and dissolved in 50% methanol, followed by centrifugation at 13,000 g for 30 min and membrane filtration for UPLC-MS analysis (G2-XS QTof, Waters). The detailed procedure was performed as described previously[9] with minor modifications. A sample comprising 2 μL of the solution was injected into the UPLC column (2.1×100 mm ACQUITY UPLC BEH C18 column containing 1.7 μm particles) with a flow rate of 0.4 mL/min. Buffer 1 (0.1% formic acid in water) and Buffer 2 (0.1% formic acid in acetonitrile) were used for the gradient elution (5% Buffer 2 for 1 min, 5-95%; Buffer 2 for 11 min, and 95% Buffer 2 for 2 min).

Mass spectrometry was performed in MS acquisition mode (electrospray source in positive ion mode) and the selected mass range was 100-1200 m/z. Leucine-enkephalin (m/z 556.2771) was used for recalibrating the lock mass option. The

ionization parameters were as follows: 2.5 kV for the capillary voltage, 40 V for the sample cone, 120 °C for the source temperature, and 800 °C for the desolvation gas temperature. MassLynx 4.1 was used for data collection and processing. Thiamine: 265.11 m/z, HET: 144.05 m/z, pyrimidine component: 404.18 m/z[10].

Quantitative PCR analysis

The total DNA of myxobacterial strain and *Phytophthora* from the co-culture assay and soil samples was extracted using a soil DNA extraction kit (FastDNA SPIN Kit, MP Biomedicals, USA). For analysis of the biomass of strains EGB and *Phytophthora*, specific pairs of DNA primers were designed based on the strain EGB *gluM* gene encoding β-1,6-glucanase[11] and *Phytophthora Actin* genes[12] or ITS sequences[13], respectively (Supplementary Table 3). All real-time quantitative PCR amplifications were run on the Applied Biosystems 7500 Real-Time PCR system using the AceQ Universal SYBR qPCR Master Mix (Vazyme, China). The biomasses of the strain EGB and *Phytophthora* were calculated from gene copy numbers using the 10-fold serial dilution method.

RNA-seq

For analysis of the cellular metabolic response to CcThi1 treatment, 0.36 and 0.71 nM of CcThi1 were added to the liquid 10% V8 culture, and a 0.2×0.2 cm agar block was cut from the growing edge of the P6497 colony and transferred into the liquid medium. A liquid culture in 10% V8 medium without CcThi1 was used as control. After incubation at 25 °C for 60 h, the mycelia were collected for total RNA isolation using a Spin Column Fungal Total RNA Purification Kit (Sangon, China) according to the

manufacturer's instructions. Paired-end read libraries were constructed using the NEBNext Ultra Directional RNA Library Prep Kit and sequenced on an Illumina HiSeq™ 2500/4000 platform (Gene Denovo Biotechnology Co. Ltd, Guangzhou, China). The produced raw sequencing data have been deposited at the NCBI SRA database (BioProject accession number: PRJNA909170). The reads were mapped to the *P. sojae* genome v1.1 (https://mycocosm.jgi.doe.gov/Physo1_1/Physo1_1.home.html) using HISAT2 with default parameters[14]. By using the Cuffdiff 2[15], the expression of each *P. sojae* gene was calculated by normalizing it to the fragment per kilobase of exon per million mapped reads (FPKM) value, and differentially expressed genes (DEGs) were identified. Genes with at least two fold change and q-value $\leqslant$ 0.05 were considered DGEs between two samples. Kyoto Encyclopedia of Genes and Genomes (KEGG) pathway enrichment analysis of DEGs were performed using the "KEGG enrichment analysis" tool embedded in the OmicsShare platform (https://www.omicshare.com/).

Supplementary Tables:

Supplementary Table 1 List of bacterial strains used in the study.

Strains	Relevant genotype or phenotype	Culture conditions	Source
**Myxobacteria**			
Corallococcus sp. EGB	Wild type (WT) from seaside soil (CCTCC No. M2012528)	VY/4 or LBS, 30 °C	Lab collection
0929	WT, *Myxococcus* sp. 0929	VY/4, 30 °C	Lab collection
0939	WT, *Myxococcus* sp. 0939	VY/4, 30 °C	Lab collection
0952	WT, *Myxococcus* sp. 0952	VY/4, 30 °C	Lab collection
1022	WT, *Corallococcus* sp. 1022	VY/4, 30 °C	Lab collection
SDU8	WT, *Archangium* sp. SDU8	VY/4, 30 °C	Lab collection
1404	WT, *Cystobacter* sp. 1404	VY/4, 30 °C	Lab collection
DK1622	Model strain, *Myxococcus xanthus*	CYE, 30 °C	D. Kaiser, Stanford University[16]
CL1003	Δ *MXAN_4523* in DK1622	CYE, 30 °C	This study
CL1004	CL1003, P_{pliA}- *ccthi1*, Kan^r, complemented with *ccthi1*	CYE, 30 °C	This study
CL1005	CL1003, P_{pliA}- *MXAN_4523*, Kan^r complemented with *MXAN_4523*	CYE, 30 °C	This study
Escherichia coli			
DH5α	Host strain for cloning vectors	LB, 37 °C	Life Technologies
BL21 (DE3)	Host strain for protein expression	LB, 37 °C	Life Technologies
K-12 Δ*thiE*	Knockout of *thiE* in *E. coli* K-12	LB or MM[2]	Horizon Discovery
Pseudomonas syringae	WT	LB, 30 °C	Lab collection
Dickeya solani	WT	LB, 30 °C	Lab collection
Bacillus subtilis	WT	LB, 30 °C	Lab collection

Supplementary Table 2 List of fungi and oomycetes used in this study.

strains	Culture conditions	Source
**Fungi**		
Magnaporthe oryzae Guy11	PDA medium, 28 °C	Lab collection
Fusarium oxysporum	PDA medium, 28 °C	Lab collection
Botrytis cinerea	PDA medium, 28 °C	Lab collection
Fusarium graminearum	PDA medium, 28 °C	Lab collection
Trichoderma harzianum	PDA medium, 28 °C	Lab collection
Pichia pastoris	YPD medium, 28 °C	Lab collection
Saccharomyces cerevisiae	YPD medium, 28 °C	Lab collection
BY4743 Δthi6	Knockout of *THI6* in *S. cerevisiae BY4743*, YPD or Delft-1[a]	HorizonDiscovery
**Oomycetes**		
Phytophthora sojae P6497	10% V8[12], 25 °C	Lab collection
GFP-labelled *Phytophthora sojae* P6497	10% V8, 25 °C	Lab collection
Phytophthora capsici	10% V8, 25 °C	Lab collection
Phytophthora parasitica	10% V8, 25 °C	Lab collection
Phytophthora infestans	Rye sucrose agar (RSA) with 10% V8[6], 18 °C	Lab collection
Pythium oligandrum	10% V8, 25 °C	Lab collection

[a] Delft-1 medium was a modified Delft medium[17] with the addition of nucleobases and amino acids (adenine, methionine, arginine, lysine, phenylalanine, tryptophan, tyrosine, and uracil: 20 mg/L; leucine: 30 mg/L; histidine: 10 mg/L; threonine: 350 mg/L; and serine: 375 mg/L).

Supplementary Table 3 List of the primers used in the study.

**Primer**	**Sequence (5'-3')**	**Function**
CcThi1-F	gctgatatcggatccgaattcATGTGCTCGGATCCTTCCG	Expression of CcThi1 from strain EGB without SP
CcThi1-R	cgagtgcggccgcaagcttTCAATGATGATGATGATGAT GCTGGAGCTGTTCGAGCAG	Expression of CcThi1 from strain EGB without SP
SP-CcThi1-F	gctgatatcggatccgaattcATGAATTGGAGAGCCCTGA TCTG	Expression of CcThi1 from strain EGB with SP
SP-CcThi1-R	cgagtgcggccgcaagcttTCAATGATGATGATGATGAT GCTGGAGCTGTTCGAGCAG	Expression of CcThi1 from strain EGB with SP
MxThi1-F	gctgatatcggatccgaattcATGTGCTCGGACCCCGAA	Expression of MxThi1 from DK1622 without SP
MxThi1-R	tcgagtgcggccgcaagcttCTAATGATGATGATGATGAT GCTGCAGCGCGTCCATCA	Expression of MxThi1 from DK1622 without SP
AcThi1-F	gctgatatcggatccgaattcATGTGCGACACGTCGGAGC	Expression of AcThi1 from *Archangium* sp. SDU8 without SP
AcThi1-R	CtcgagtgcggccgcaagcttTCAATGATGATGATGATGA TGGGGGGCCTTCGTGGT	Expression of AcThi1 from *Archangium* sp. SDU8 without SP
CyThi1-F	gctgatatcggatccgaattcATGTGTGGTGGGAAGCCACC	Expression of CyThi1 from *Cystobacter* sp. 1404 without SP
CyThi1-R	CtcgagtgcggccgcaagcttCTAATGATGATGATGATGA TGCTGGAGCGCGGCCTG	Expression of CyThi1 from *Cystobacter* sp. 1404 without SP
CbThi1-F	gctgatatcggatccgaattcATGTTTTCAGGGGATGAACCC	Expression of CbThi1 from *Clostridium botulinum* without SP
CbThi1-R	ctcgagtgcggccgcaagcttTCATTGTTGTAAATATTCAG TAATAATTTTCT	Expression of CbThi1 from *Clostridium botulinum* without SP
Δ4523-UF	aaaacgacggccagtgaattcTAACACTCGTACTCACGAGG AATGA	Amplification of upstream homologous arm for deletion of *MXAN_4523* in DK1622
Δ4523-UR	TGGCTTTGTCTGTATATTGTCGTCATGCGTCACGGC	Amplification of upstream homologous arm for deletion of *MXAN_4523* in DK1622

Δ4532-DF	cgtgacgcatgacgacaATATACAGACAAAGCCATCCAGCA	Amplification of downstream homologous arm for deletion of *MXAN_4523* in DK1622
Δ4523-DR	gaccatgattacgccaagcttAGTCCCTTGGTCAGACCTTCCA	Amplification of downstream homologous arm for deletion of *MXAN_4523* in DK1622
CcThi1-cF	tctctgaggacccccctctagaATGAATTGGAGAGCCCTGATCTG	*ccthi1* complementation in strain CL1003, *pilA* promoter
CcThi1-cR	cgacggccagtgccaagcttTCAATGATGATGATGATGATGCTGGAGCTGTTCGAGCA	*ccthi1* complementation in strain CL1003, *pilA* promoter
4523-cF	tctctgaggacccccctctagaTTGATACCGCTCCCGCCG	*MXAN_4523* complementation in strain CL1003, *pilA* promoter
4523-cR	cgacggccagtgccaagcttCTAATGATGATGATGATGATGCTGCAGCGCGTCCATCA	*MXAN_4523* complementation in strain CL1003, *pilA* promoter
EGB-F	TCATCATCGGCACTGTCATC	Quantitative PCR analysis for biomass of strain EGB
EGB-R	GGATGGTGCGGTTGAGGAGC	Quantitative PCR analysis for biomass of strain EGB
PS-F1	CGAAATTGTGCGCGACATCAAG	Quantitative PCR analysis for biomass of P6497
PS-R1	GGTACCGCCCGACAGCACGAT	Quantitative PCR analysis for biomass of P6497
PS-F2	GCTGTCTGTCGATGTCAAAG	Quantitative PCR analysis for biomass of P6497 in soil
PS-R2	AGGAGGACCCAAACCAAT	Quantitative PCR analysis for biomass of P6497 in soil
PC-F1	AGGAGATGGCCAAGTTAGC	Quantitative PCR analysis for biomass of *P. capsici*
PC-R1	CCGACTCATCATACTCGG	Quantitative PCR analysis for biomass of *P. capsici*
PP-F1	CACGTACACATGCCCGAGAC	Quantitative PCR analysis for biomass of *P. parasitica*
PP-R1	TTCCCATGTAGGCCGAGTATTC	Quantitative PCR analysis for biomass of *P. parasitica*

Note: Lowercase letters denote homologous sequences for plasmid construction. SP: signal peptide.

Supplementary Table 4 List of the plasmids used in this study.

**Plasmids**	**Characteristics**	**Source**
pET-29a (+)	Expression vector; Kan^r	TaKaRa
pET-29a (+)-*ccthi1*	pET-29a (+) derivative carrying *ccthi1* without signal peptide; Kan^r	This study
pET-29a (+)- *sp- ccthi1*	pET-29a (+) derivative carrying *ccthi1* with signal peptide; Kan^r	This study
pET-29a (+)-*sp-ccthi1*$^{C148A; E269A}$	pET-29a (+) derivative carrying *ccthi1*$^{C148A; E269A}$ without signal peptide; Kan^r	This study
pET-29a (+)- *MXAN_4523*	pET-29a (+) derivative carrying *MXAN_4523* without signal peptide; Kan^r	This study
pSWU19	Site specific integration vector with Mx8 *attB* site; Kan^r	Lab collection
P_{pilA} - *ccthi1*- pSWU19	*pilA* promoter, *ccthi1* gene cloned in pSWU19; Kan^r	This study
P_{pilA} - *MXAN_4523*- pSWU19	*pilA* promoter, *MXAN_4523* gene cloned in pSWU19; Kan^r	This study
pBJ113	Gene replacement vector with KG cassette; Kan^r	Lab collection
pCL03	*MXAN_4523* in-frame deletion PCR product cloned into pBJ113; Kan^r	This study

Supplementary Table 5 Identification of the purified active protein using NanoLC-ESI-MS/MS.

**Hits**	**Protein Mass**	**No. of Unique Peptide**	**Peptide sequence**	**GenBank accession**	**Relative Abundance**	**Predicted function**
1	43855.8	6	GGRPYPNQR<br>SLIAFSKDAPAGTR<br>KALQAALLEQLQ<br>IGGETYGVPTYLCTNVVYSR<br>WIQPVALEAGVVHPAAEEAVR<br>AVLFPYIPDSAGDGFASLEQR	WP_223633232.1	32.9%	extracellular solute-binding protein
2	64889.61	1	APLLGQQALEAKLVDGLRYR	WP_223640577.1	49.9%	signal peptide peptidase SppA
3	54127.17	3	FFEPGGNHGASIQGLPEADQAAALER<br>IVQAFKPLFPGK<br>ILLVYGENDPWSTGAFSVSAR	WP_223642068.1	2.9%	S28 family serine protease
4	42631.36	1	RAATMGGPKR	WP_223638619.1	14.3%	YhcG family protein

Note: Protein identification by MS/MS database searches was performed using the NCBI reference genome of *Corallococcus* sp. strain EGB (Genome ID: 17964).

Supplementary Table 6 Representative thiamine-producing bacteria from rhizosphere

Strain Number	Genera	Thiamine production	Strain Number	Genera	Thiamine production	Strain Number	Genera	Thiamine production
2801	*Escherichia* (T-1)	+++	1500	*Paenarthrobacter*	+	1832	*Microbacterium*	-
2299	*Acidovorax*	+	1466	*Paenibacillus*	++	2311	*Microbacterium*	-
1414	*Arthrobacter*	+	2326	*Pedococcus*	+	2317	*Microbacterium*	-
1423	*Arthrobacter*	+	1426	*Peribacillus*	+	2309	*Mycolicibacterium*	-
1437	*Arthrobacter*	+	1417	*Pseudarthrobacter*	+	2310	*Mycolicibacterium*	-
1488	*Arthrobacter*	+	1438	*Pseudarthrobacter*	+	1706	*Neorhizobium*	-
1502	*Arthrobacter*	+	1497	*Pseudarthrobacter*	+	1825	*Novosphingobium*	-
1830	*Arthrobacter*	+	1826	*Pseudarthrobacter*	+	1859	*Pseudomonas*	-
1425	*Bacillus*	+++	1849	*Pseudarthrobacter*	+++	1495	*Pseudoxanthomonas*	-
1427	*Bacillus*	++	1412	*Pseudomonas*	++	1695	*Pseudoxanthomonas*	-
1441	*Bacillus*	+++	1419	*Pseudomonas*	+++	1842	*Ralstonia*	-
1456	*Bacillus*	++	1434	*Pseudomonas*	+++	2334	*Ramlibacter*	-
1478	*Bacillus*	+++	1499	*Pseudomonas*	+	1482	*Rhizobium*	-
1676	*Bacillus*	++	1833	*Pseudomonas*	+	1685	*Rhizobium*	-
1416	*Brevundimonas*	+	1820	*Ralstonia*	+	1699	*Sphingomonas*	-
1447	*Brevundimonas*	+	1458	*Rhizobium*	++	1688	*Sphingomonas*	-
1503	*Brevundimonas*	+++	1421	*Sphingobacterium*	+	2318	*Sphingopyxis*	-
1465	*Caulobacter*	++	2293	*Sphingomonas*	+	1694	*Stenotrophomonas*	-
2321	*Cellulomonas*	+	1433	*Stenotrophomonas*	+++	1697	*Stenotrophomonas*	-
1741	*Chitinophaga*	+	1501	*Stenotrophomonas*	+	1704	*Stenotrophomonas*	-
1822	*Chitinophaga*	+	1702	*Stenotrophomonas*	+	1719	*Streptomyces*	-
1431	*Chryseobacterium*	+	1797	*Streptomyces*	++	1798	*Streptomyces*	-
1504	*Comamonas*	++	1808	*Streptomyces*	+	1799	*Streptomyces*	-
1853	*Dyadobacter*	+	2308	*Streptomyces*	++	2305	*Streptomyces*	-
1477	*Dyella*	+	1413	*Agrobacterium*	-	2314	*Streptomyces*	-
1819	*Massilia*	++	1818	*Arthrobacter*	-	2333	*Streptomyces*	-
1707	*Mesorhizobium*	+	1444	*Bacillus*	-			
1445	*Microbacterium*	+	1449	*Bacillus*	-			
1446	*Microbacterium*	+++	1485	*Bacillus*	-			
1462	*Microbacterium*	+++	1496	*Brucella*	-			
1486	*Microbacterium*	+	1831	*Chitinophaga*	-			
1701	*Microbacterium*	++	1834	*Chitinophaga*	-			
1824	*Microbacterium*	+	1430	*Chryseobacterium*	-			
1829	*Microbacterium*	+	1703	*Cytobacillus*	-			
1841	*Microbacterium*	+	1816	*Dyadobacter*	-			
2307	*Microbacterium*	+	2298	*Fulvimonas*	-			
1821	*Moraxella*	+	1429	*Kosakonia*	-			
2316	*Mycolicibacterium*	+	1801	*Kribbella*	-			
1705	*Neorhizobium*	+	1696	*Luteimonas*	-			
2315	*Nocardioides*	+	1420	*Microbacterium*	-			
2327	*Nocardioides*	+	1708	*Microbacterium*	-			

Supplementary Figures:

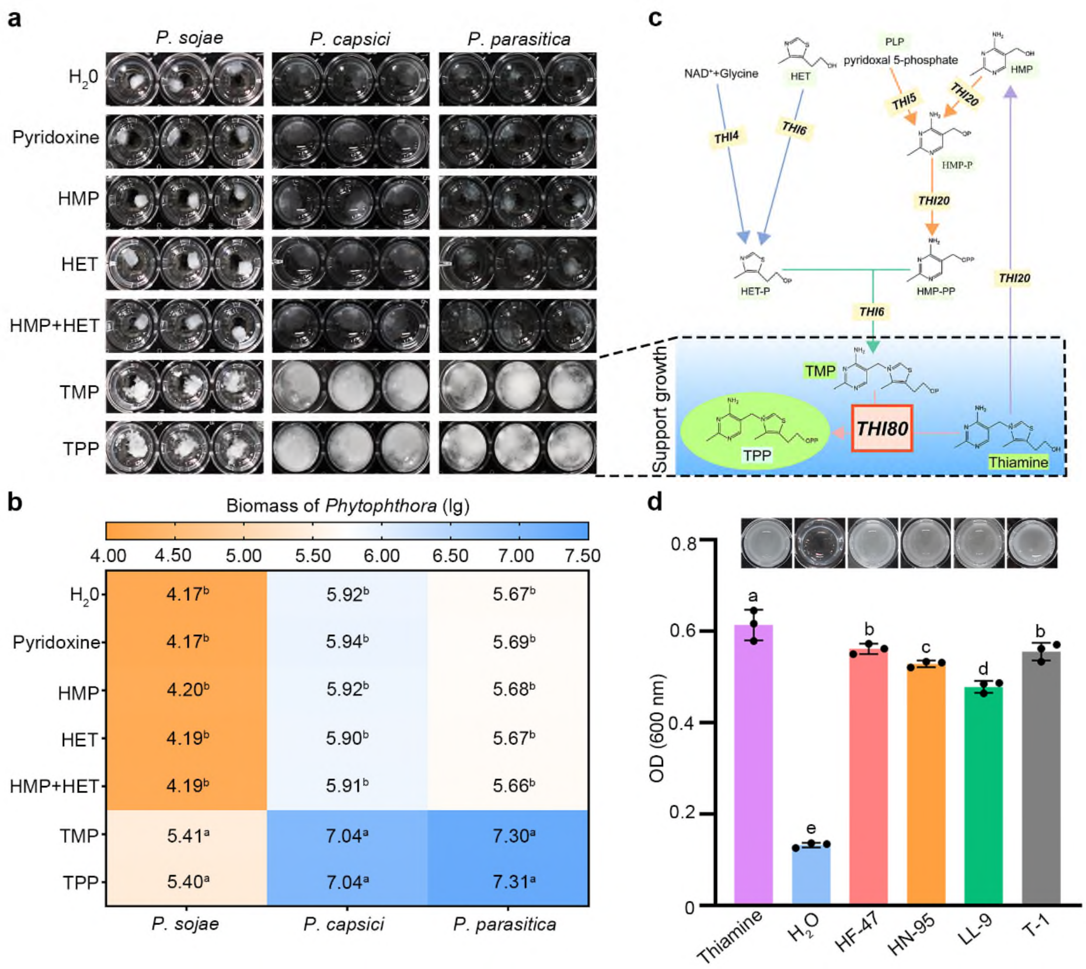

Supplementary Fig. 1 Members of the genus *Phytophthora* are thiamine auxotrophs and require exogenous sources of thiamine for growth. a, b, *P. sojae*, *P. capsici* and *P. parasitica* were cultured in liquid P1 medium with the addition of vitamins and different precursors. Agar discs were removed from the edge of an actively growing *Phytophthora* colony and transferred to the center of 48-well plates containing 490 µL of defined medium and 2 µM vitamins and precursors. The precursors HMP and HET are derived from the thiamine biosynthesis pathway. The growth of P6497 was observed after 10 d of incubation at 25 °C using a stereomicroscope (Nikon SMZ-10), and the abundance of *Phytophthora* was measured by qPCR **(b)**. The data represent the

means ± SEM (n = 3 biological independent replicates). **c,** Thiamine biosynthesis pathway of *Saccharomyces cerevisiae*[18]. *TH180* (Red boxes) in *Saccharomyces cerevisiae* is responsible for the biosynthesis of TPP from TMP and thiamine, and its homolog is present in *Phytophthora* (*P. sojae* gene ID: 20663896; *P. infestans* gene ID: 9462311) according to comparative genome sequence analysis, but there are no homologs of *TH14*, *TH15*, *THI6* and *THI20*. TMP: thiamine monophosphate; TPP: thiamine pyrophosphate; HMP: hydroxymethylpyrimidine; HET: hydroxyethylthiazole. **d,** Secreted thiamine from soybean root exudates and soil microbes supports the growth of *E. coli* K-12 Δ*thiE* deficient in thiamine synthesis. The above picture shows the growth of strain K-12 Δ*thiE* in 48-well plates with the addition of root exudates (0.1 mg/mL) and supernatant of thiamine-producing strain T-1 (5 mg/mL). The plates were cultured at 37 °C for 24 h, and the growth of strain K-12 Δ*thiE* was determined by measurement of the OD_{600}. HF-47, variety Hefeng-47; HN-95, variety Huning 95-1; LL-9, variety Lvling 9; T-1, thiamine-producing strain T-1 that was isolated from rhizosphere soil (Supplementary Table 6). The data represent the means ± SEM (n = 3 biological independent replicates), and means within columns followed by a different letter are significantly different in **b** and **d** ($p < 0.05$, one-way ANOVA, Duncan's multiple range test). Source data are provided as a Source Data file.

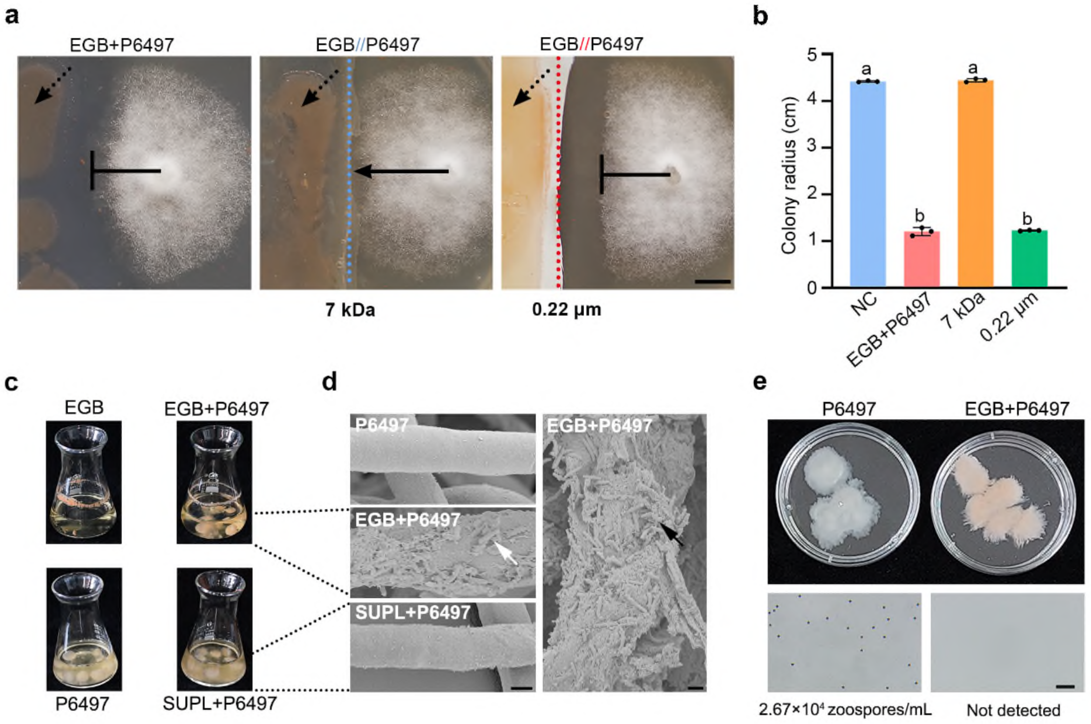

Supplementary Fig. 2 *Corallococcus* sp. strain EGB inhibits the growth and zoospores production of P6497 from co-culture. a, Co-culture of strain EGB and P6497 on 10% V8 plates with (EGB// P6497) or without (EGB+P6497) membrane separation. The blue dotted line indicates the semi-permeable membrane (7 kDa molecular weight cutoff), red dotted line indicates the membrane filter (0.22 μm pore size), and dotted arrow indicates the location of strain EGB; → indicates the normal growth, ├ indicates the inhibited growth. Scale bars: 5 mm. **b,** Colony diameter measurement of P6497 from the co-culture assay (**a).** All the data represent the means ± SEM (n = 3 biological independent replicates). Means within columns followed by a different letter are significantly different ($p < 0.05$, one-way ANOVA, Duncan's multiple range test). **c**, Growth state of strain EGB and P6497 in liquid co-culture. Co-culture assay of P6497 (0.1 g wet weight) and strain EGB (10^5 cells/mL) or its secreted SUPL (10 mg/mL) was performed in 25 mL of TV medium at 30 °C and 180 rpm for 3

d. Monocultures of strain EGB or P6497 were used as control. **d,** Representative SEM images of P6497 mycelia from the co-culture assay with strain EGB or SUPL, scale bars: 2 μm. White arrows indicate attachment sites of strain EGB cells, black arrows indicate the biofilm formation of strain EGB on mycelia. SUPL: Lyophilized cell-free supernatant of strain EGB. **e**, Effect of strain EGB incubation on the zoospores production of P6497. Scale bars: 100 μm. Source data are provided as a Source Data file.

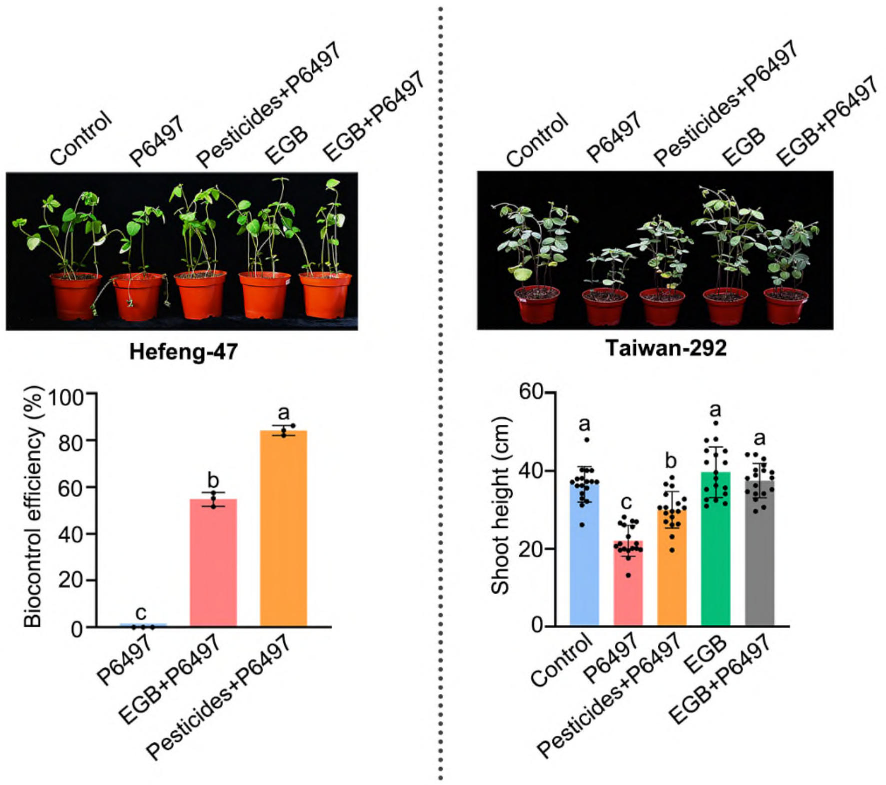

Supplementary Fig. 3 ***Corallococcus*** **sp. EGB controls the stem rot disease in soybean seedlings.** Soybean (susceptible cultivar Hefeng-47 and resistant cultivars Taiwan-292) growth in a greenhouse pot experiment following treatment with strain EGB and pesticide treatments against P6497 infection. Five treatments were designed: Control, setup of soybean seedlings prepared without any treatment; P6497, soybean seedlings incubated with *P. sojae*; EGB+P6497, mixture of *P. sojae* and EGB (5 mL, 10^8 cells/mL); pesticide+P6497, mixture of *P. sojae* and pesticide. The chemical pesticide was a mixture of hymexazol and metalaxyl at the recommended dosage (2,000-fold dilution). Biocontrol efficiency and shoot height were measured in the pot experiments. All the data represent the means ± SEM (n = 3 biological independent replicates for biocontrol efficiency; n = 18 biological independent replicates for shoot height). Means within columns followed by a different letter are significantly different ($p < 0.05$, one-way ANOVA, Duncan's multiple range test). Source data are provided

as a Source Data file.

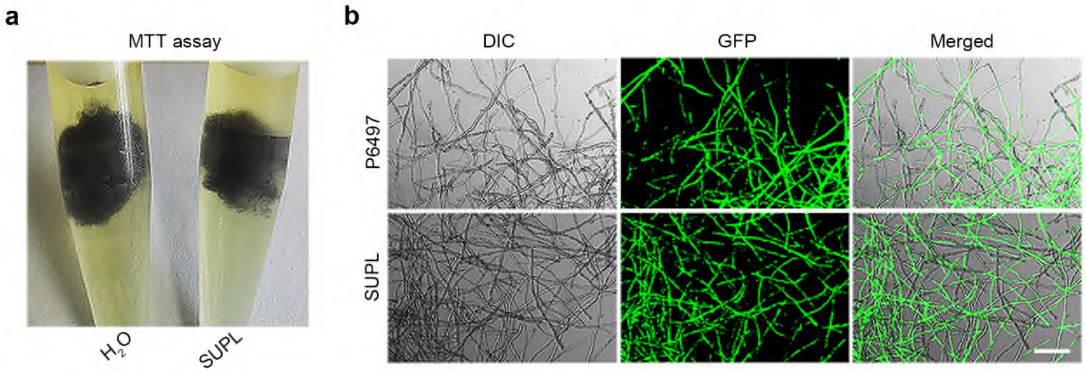

Supplementary Fig. 4 Treatment with SUPL from *Corallococcus* sp. EGB fails to kill P6497. a, MTT staining of P6497 mycelia with and without SUPL treatment. **b**, Microscopic analysis of SUPL-treated GFP-labelled P6497 mycelia. The reaction systems containing SUPL (10 mg/mL) and GFP-labelled P6497 mycelia were incubated at 25 °C for 24 h. Scale bar, 100 µm.

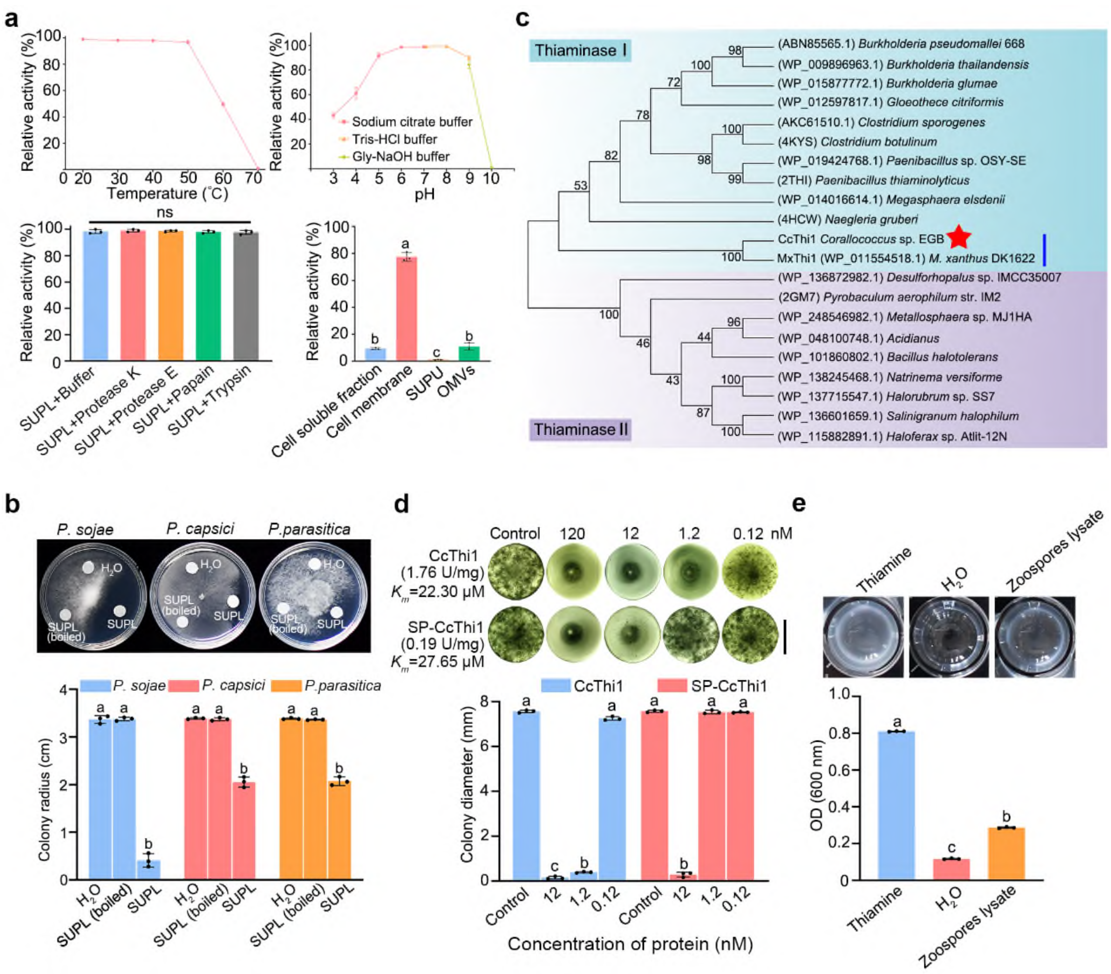

Supplementary Fig. 5 Biochemical properties of SUPL and CcThi1 and their potential inhibitory activities against *Phytophthora*. a, Effects of temperature, pH and protease (1 mg/mL) on the activity of SUPL, and the distribution of active fractions in different cellular components. SUPU denotes supernatant without OMVs. **b,** Effects of SUPL (10 mg/mL, 5 µL) on the growth of *P. sojae*, *P. capsici*, and *P. parasitica*. The growth inhibition was quantified by measurement of colony diameter. H_2O and heat-inactivated SUPL were used as control. **c,** Phylogenetic tree analysis of CcThi1 and related sequences of thiaminase I and thiaminase II using the neighbor-joining method. The red star indicates CcThi1 and the blue line indicates the branch where the thiaminase I homologs from myxobacteria are located. **d,** Comparison of enzymatic and

antimicrobial activity of CcThi1 and SP-CcThi1 against *Phytophthora*. The enzyme activity was determined using thiamine and 4-NTP as the substrate and nucleophile, respectively. The colony diameter of P6497 after treatment with CcThi1 (without signal peptide) and SP-CcThi1 (with signal peptide) was measured. Scale bar, 5 mm. **e,** Growth of thiamine auxotrophic strain K-12 Δ*thiE* in 48 well-plates with the addition of thiamine (1 μM) and lysate of P6497 zoospores (2×10^7 zoospores). The thiamine content was determined using a bioassay method. The plates were cultured at 37 °C and 180 rpm for 20 h. The growth of strain K-12 Δ*thiE* was determined by measuring the OD_{600}. All the data represent the means ± SEM (n = 3 biological independent replicates). Means within columns followed by a different letter are significantly different in **a**, **b**, **d** and **e** ($p < 0.05$, one-way ANOVA, Duncan's multiple range test), ns represents non-significant difference in **a**. Source data are provided as a Source Data file.

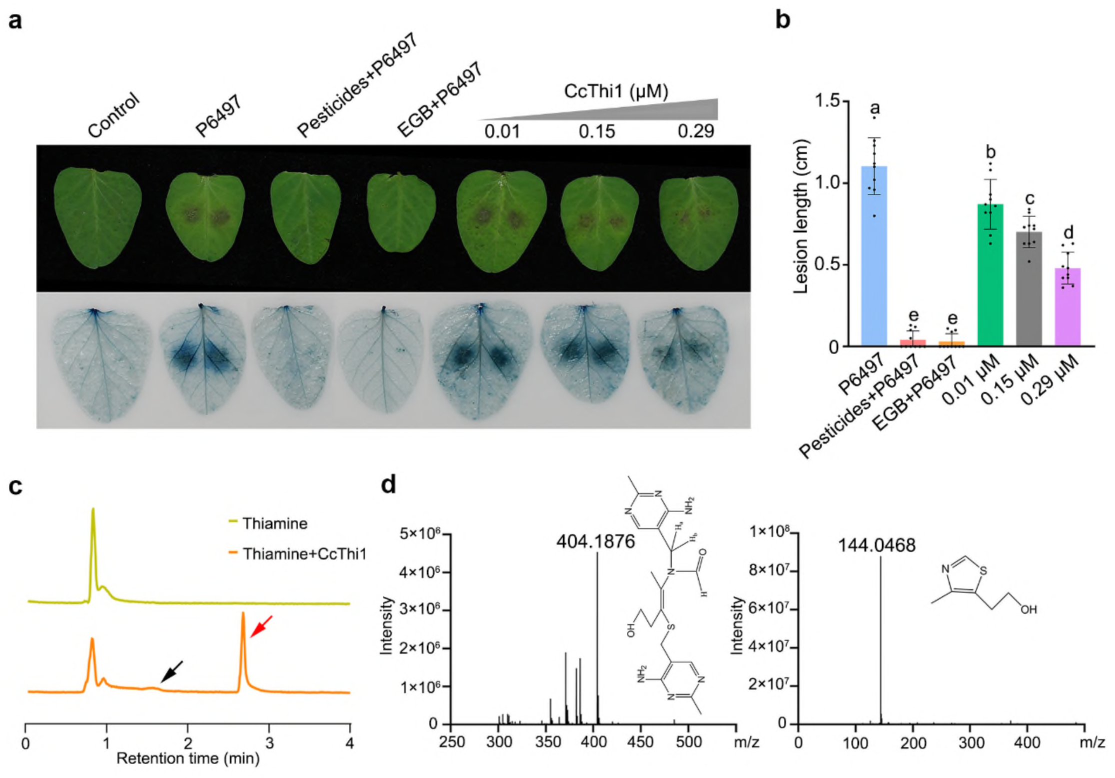

Supplementary Fig. 6 Soybean leaf infection and thiamine decomposition assay. a, b, *In vitro* leaf infection experiment. Representative soybean leaves (Hefeng-47) after 36 h inoculation with P6497, EGB+P6497, pesticide+P6497, and thiaminase I CcThi1 at different concentrations (0.01, 0.15 and 0.29 μM) were present, and All the leaves were stained with lactophenol trypan blue and decolorized with chloral hydrate (**a**) The corresponding lesion diameters were measured (**b**). The data represent the means ± SEM (n = 10 biological independent replicates). Means within columns followed by a different letter are significantly different ($p < 0.05$, one-way ANOVA, Duncan's multiple range test). **c, d,** CcThi1 decomposes thiamine into thiazole and pyrimidine without nucleophile. UPLC-MS analysis of the products generated by CcThi1 was performed with thiamine as the substrate (**c**). The black arrow indicates the peak corresponding to the pyrimidine component, and the red arrow indicates the peak

corresponding to HET. The retention time and molecular weight (mass-to-charge ratio, m/z) of pyrimidine component[10] (404.18 m/z, left) and HET (144.05 m/z, right) were deduced from UPLC-MS analysis (**d**). Reaction conditions: 1.19 μM CcThi1 and 0.29 mM thiamine reacted at 25 °C for 6 h, after which the reaction was terminated at 95 °C for 5 min. Source data are provided as a Source Data file.

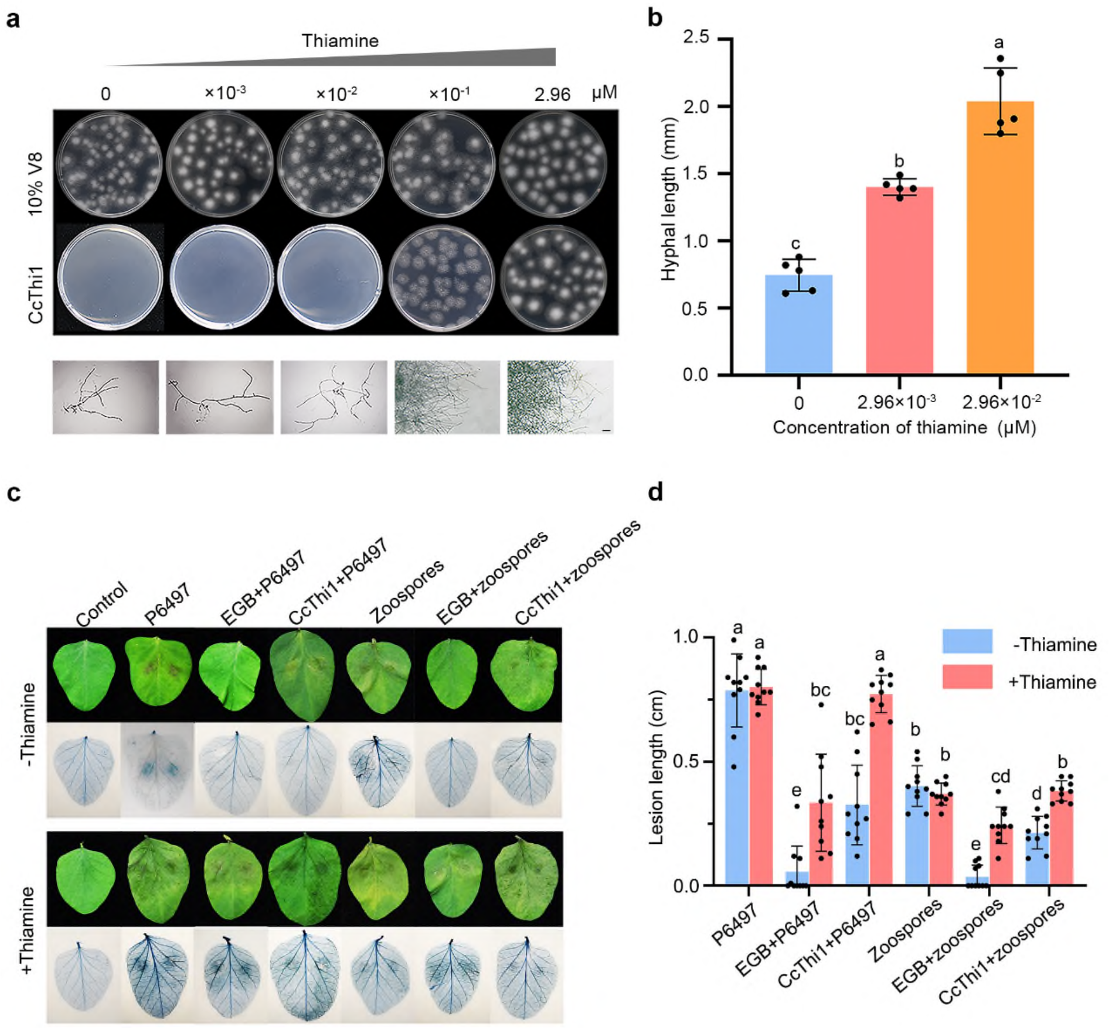

Supplementary Fig. 7 Effects of thiamine supplementation on zoospores growth and soybean infection of *Phytophthora*. a, b, Inhibition of mycelial growth on 10% V8 plates by CcThi1 (0.24 nM) with supplementation of various concentrations of thiamine (0, 2.96×10^{-3}, 2.96×10^{-2}, 2.96×10^{-1} and 2.96 μM) (**a**), and microscopic observation of mycelia after CcThi1 treatment was performed to measure the hyphal length after 7 d of incubation (**b**). The data represent the means ± SEM (n = 5 biological independent replicates). Scale bars: 100 μm. **c, d**, Effects of thiamine supplementation on infections of *Phytophthora* mycelia and zoospores toward soybean leaves. All the leaves were stained with lactophenol trypan blue and decolorized with chloral hydrate

(**c**), and the corresponding lesion diameters were measured (**d**). The data represent the means ± SEM (n = 10 biological independent replicates). Means within columns followed by a different letter are significantly different in **b** and **d** ($p < 0.05$, one-way ANOVA, Duncan's multiple range test). Source data are provided as a Source Data file.

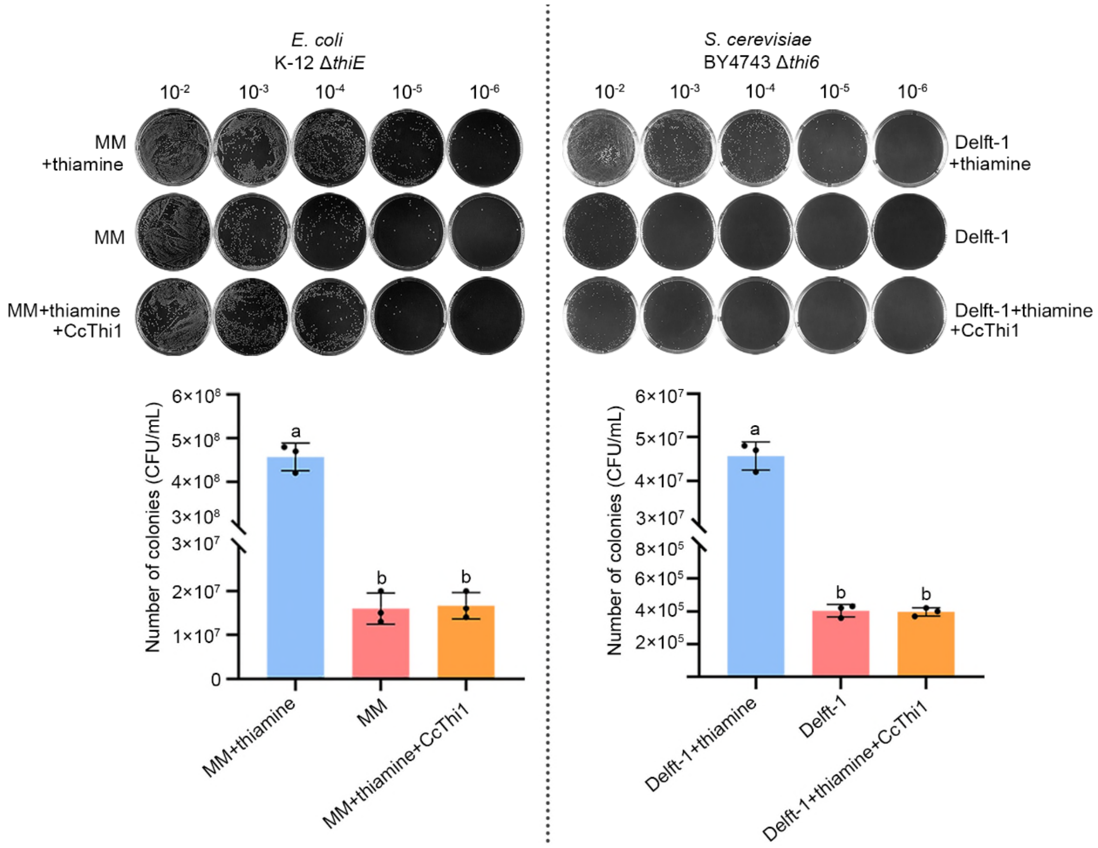

Supplementary Fig. 8 CcThi1 restricts the growth of thiamine auxotrophic *E. coli* K-12 Δ*thiE* and *S. cerevisiae* BY4743 Δ*thi6*. Effects of CcThi1 (2 μM) on the growth of K-12 Δ*thiE* (10^6 cells/mL) and BY4743 Δ*thi6* (10^5 cells/mL) in MM medium and Delft-1 medium with or without thiamine (0.01 or 1 μM). The numbers of viable colonies were counted after serial dilution (10^{-2}-10^{-6}) and incubation at 30 °C for 24 h. All the data represent the means ± SEM (n = 3 biological independent replicates), and means within columns followed by a different letter are significantly different ($p < 0.05$, one-way ANOVA, Duncan's multiple range test). Source data are provided as a Source Data file.

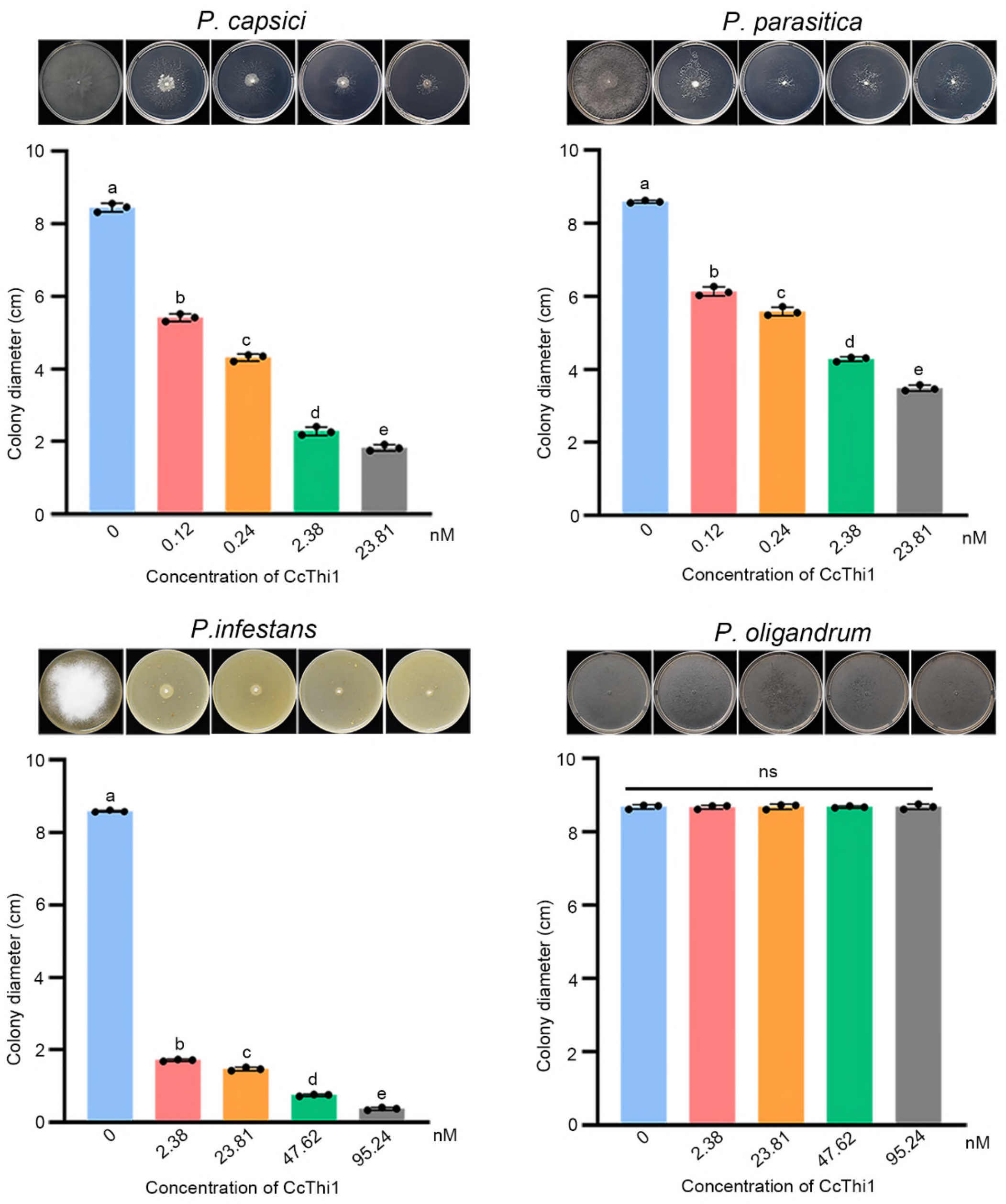

Supplementary Fig. 9 Effects of CcThi1 on the growth of oomycetes. Effects of CcThi1 at various concentrations on the growth of *P. capsica*, *P. parasitica*, *P. infestans* and *P. oligandrum*, and the growth inhibition was quantified by measuring the colony diameter. All the data represent the means ± SEM (n = 3 biological independent replicates), and means within columns followed by a different letter are significantly different ($p < 0.05$, one-way ANOVA, Duncan's multiple range test). ns represents non-significant difference. Source data are provided as a Source Data file.

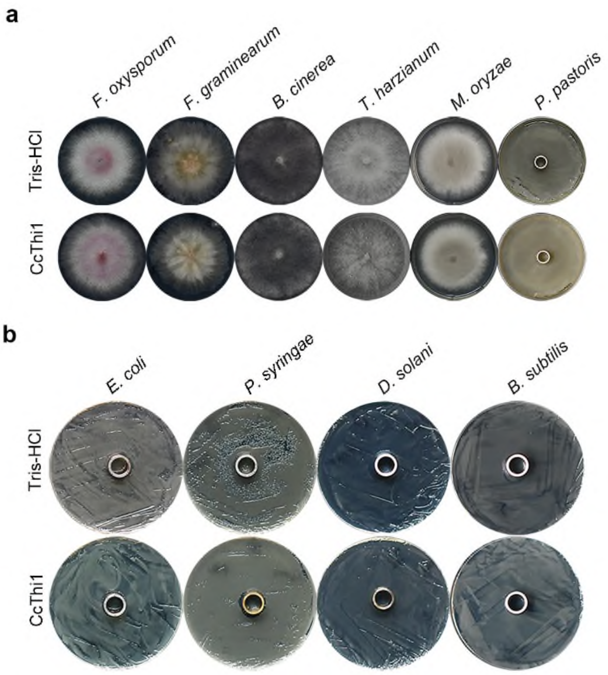

Supplementary Fig. 10 Effects of CcThi1 on the growth of fungi (a) and bacteria (b). a, For filamentous fungi, agar discs were removed from the edge of an actively growing colony and transferred to the center of plates containing 0.24 μM CcThi1, after which the plates were incubated at 28 °C for 5-7 d. **b**, For bacteria, a suspension containing 10^5 cells was spread on the plate, and an Oxford cup loaded with 200 μL of CcThi1 (11.9 μM) was placed at the center of the plate, followed by incubation at 30 °C for 2 d. Buffer: 50 mM Tris-HCl (pH 7.2).

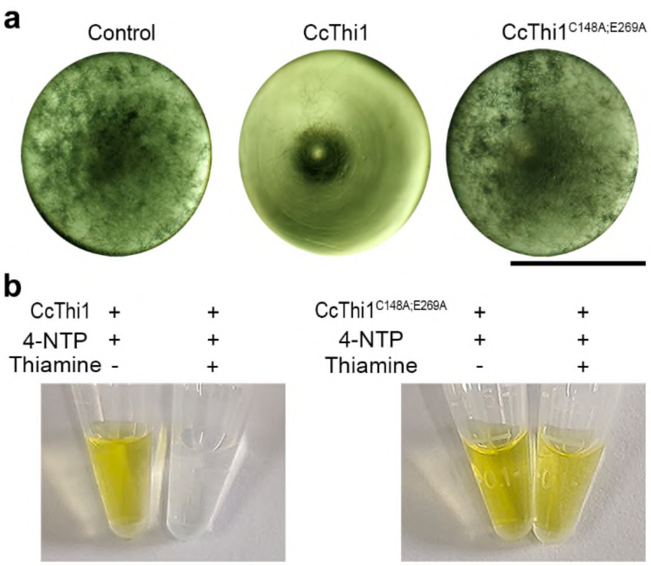

Supplementary Fig. 11 Antimicrobial effect against *Phytophthora* (a) and thiaminase I activity (b) of CcThi1 and mutant CcThi1$^{C148A;\ E269A}$. a, Agar discs were removed from the edge of an actively growing *P. sojae* colony and transferred to the center of 96-well plates containing 90 μL liquid V8 medium and 10 μL protein (100 μg/mL). The growth of *P. sojae* was observed using a stereomicroscope (Nikon SMZ-10) after 20 h at 25 °C. Scale bar, 5 mm. **b,** To identify the thiaminase activity of CcThi1 and mutant CcThi1$^{C148A;\ E269A}$, 194 μL buffer (100 mM NaCl; 50 mM phosphate buffer, pH 7.2; 10 mM TCEP; 200 μM 4-nitrothiophenol (4-NTP); 400 μM thiamine) and 6 μL protein solution (100 μg/mL) were mixed and incubated at 37 °C for 4 h.

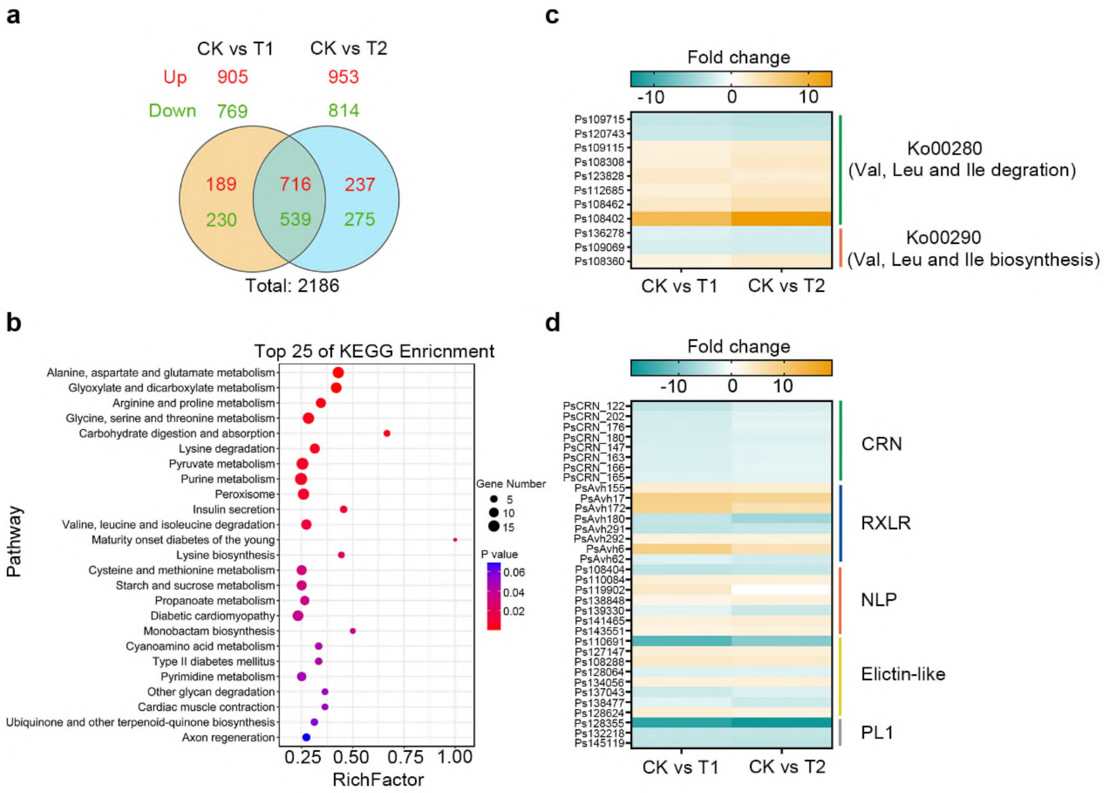

Supplementary Fig. 12 CcThi1 influences the metabolic process of P6497. a, A Venn diagram showing differentially expressed genes (DEGs) (q≤0.05, Fold change > 2 or < -2). CK, P6497 without treatment; T1, P6497 treated with 0.36 nM CcThi1; T2, P6497 treated with 0.71 nM CcThi1. **b,** Bubble plot showing results of KEGG enrichment (top 25) analysis of repeated DEGs. Transcriptional changes in genes related to the metabolic pathways of P6497 after treatment with CcThi1 are shown. **c,** Transcriptional changes in genes related to amino acid synthesis and degradation in P6497 after treatment with CcThi1. Ko00280: valine, leucine and isoleucine degradation pathways, Ko00290: valine, leucine and isoleucine synthesis pathways. **d,** Transcriptional changes in genes related to the effector and virulence factor in P6497 after treatment with CcThi1. CRN, crinkling and necrosis proteins; NLP, NEP1-like family; PL1, pectate lyase PL1. Source data are provided as a Source Data file.

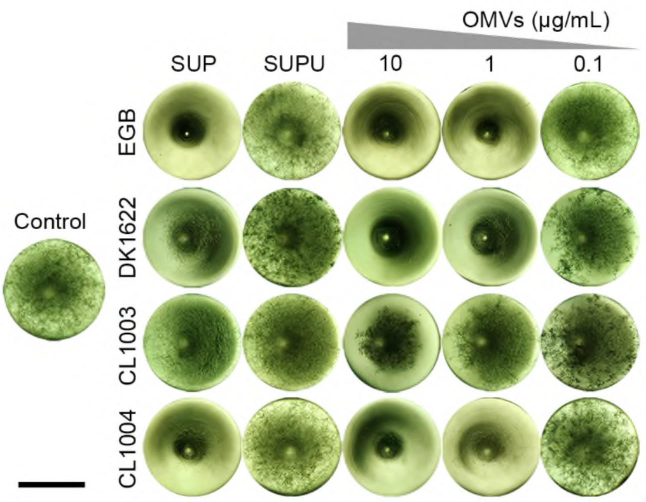

Supplementary Fig. 13 Effects of SUP, SUPL and OMVs on the growth of P6497 at various concentrations (0.1, 1 and 10 μg/mL). Agar discs were removed from the edge of an actively growing *P. sojae* colony and transferred to the center of 96-well plates containing 90 μL of liquid 10% V8 medium and 10 μL of the indicated additives. The growth of P6497 was observed using a stereomicroscope (Nikon SMZ-10) after incubation at 25 °C for 20 h. SUP, culture supernatant; SUPU, supernatant after ultracentrifugation; OMVs: outer membrane vesicles. Scale bar, 5 mm.

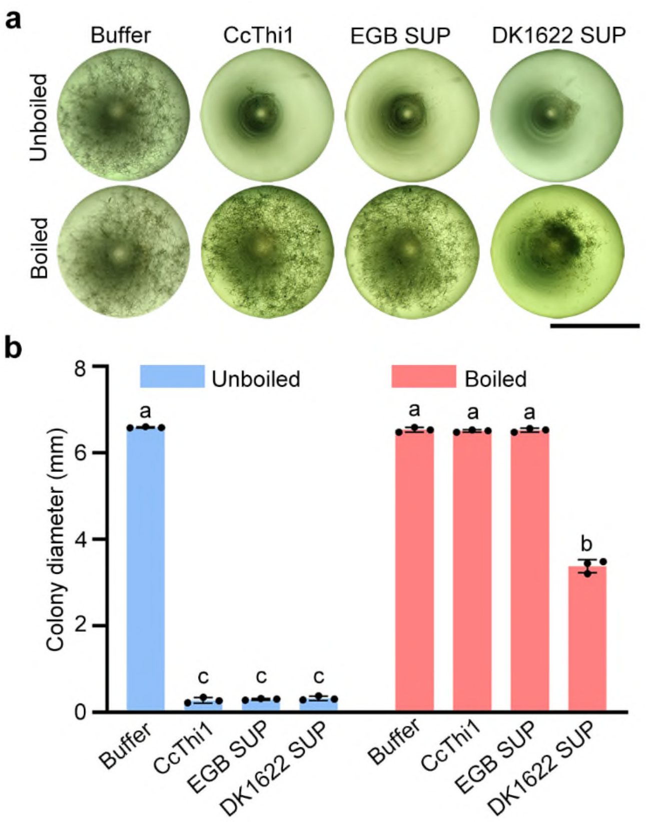

Supplementary Fig. 14 Heat stability of SUP from strains EGB and DK1622. a, Growth state of P6497 in 96 well-plates. P6497 was cultured at 25 °C for 20 h in 96 well-plates with 50 μL of 10% V8 medium and 50 μL of the indicated additives. The boiled SUP and 50 mM Tris-HCl buffer (Buffer) were used as control. The growth of P6497 was observed using a stereomicroscope (Nikon SMZ-10) after incubation at 25 °C for 20 h. Scale bar, 5 mm. **b,** The colony diameter of P6497 was measured as described above. The data represent the means ± SEM (n = 3 biological independent replicates), and means within columns followed by different letters are significantly different ($p < 0.05$, one-way ANOVA, Duncan's multiple range test). Source data are provided as a Source Data file.

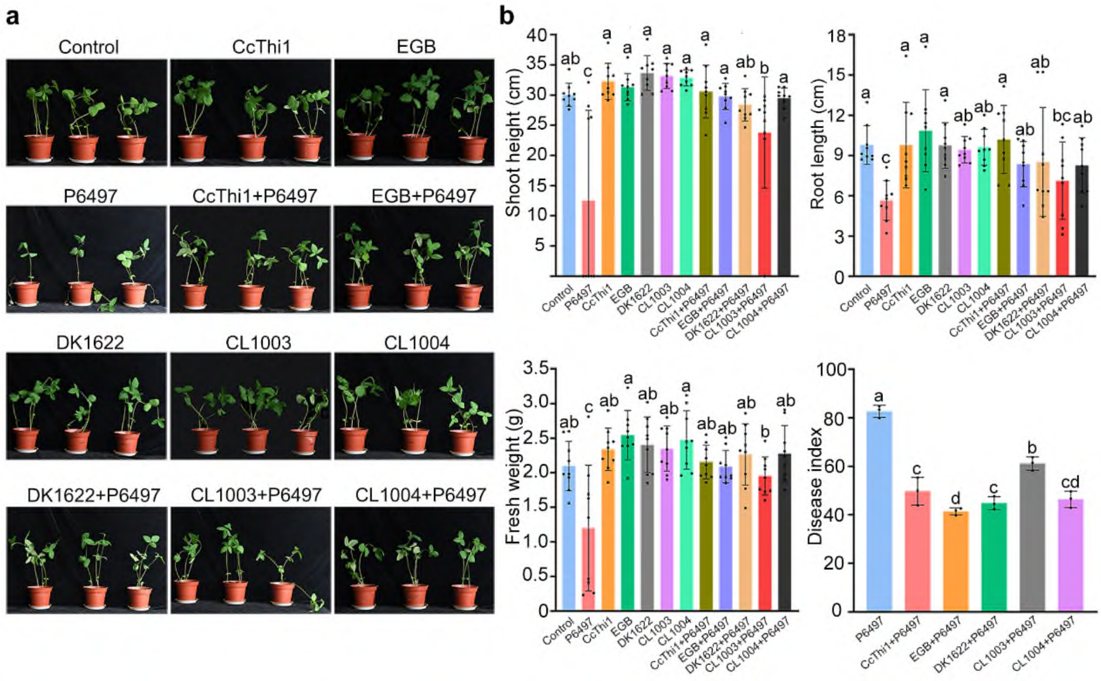

Supplementary Fig. 15 Myxobacteria and CcThi1 controls soybean root rot disease in pot experiments. a, The soybean plants were placed in a growth chamber at 25 °C and pictures of plants were taken after 21 d (Fig. 5a). The soybean roots from different pots containing P6497 agar plates were inoculated with 5 mL myxobacterial suspension (10^8 cells/mL) of strain EGB, DK1622, CL1003 and CL1004, or irrigated with CcThi1 (1.2 nmol/pot every other day). **b,** Measurement of shoot height, root length and fresh weight of soybean plants and disease index following the indicated treatments shown above. The data represent the means ± SEM (n = 9 biological independent replicates for shoot height, root length and fresh weight; n = 3 biological independent replicates for disease index), and means within columns followed by a different letter are significantly different ($p < 0.05$, one-way ANOVA, Duncan's multiple range test). Source data are provided as a Source Data file.

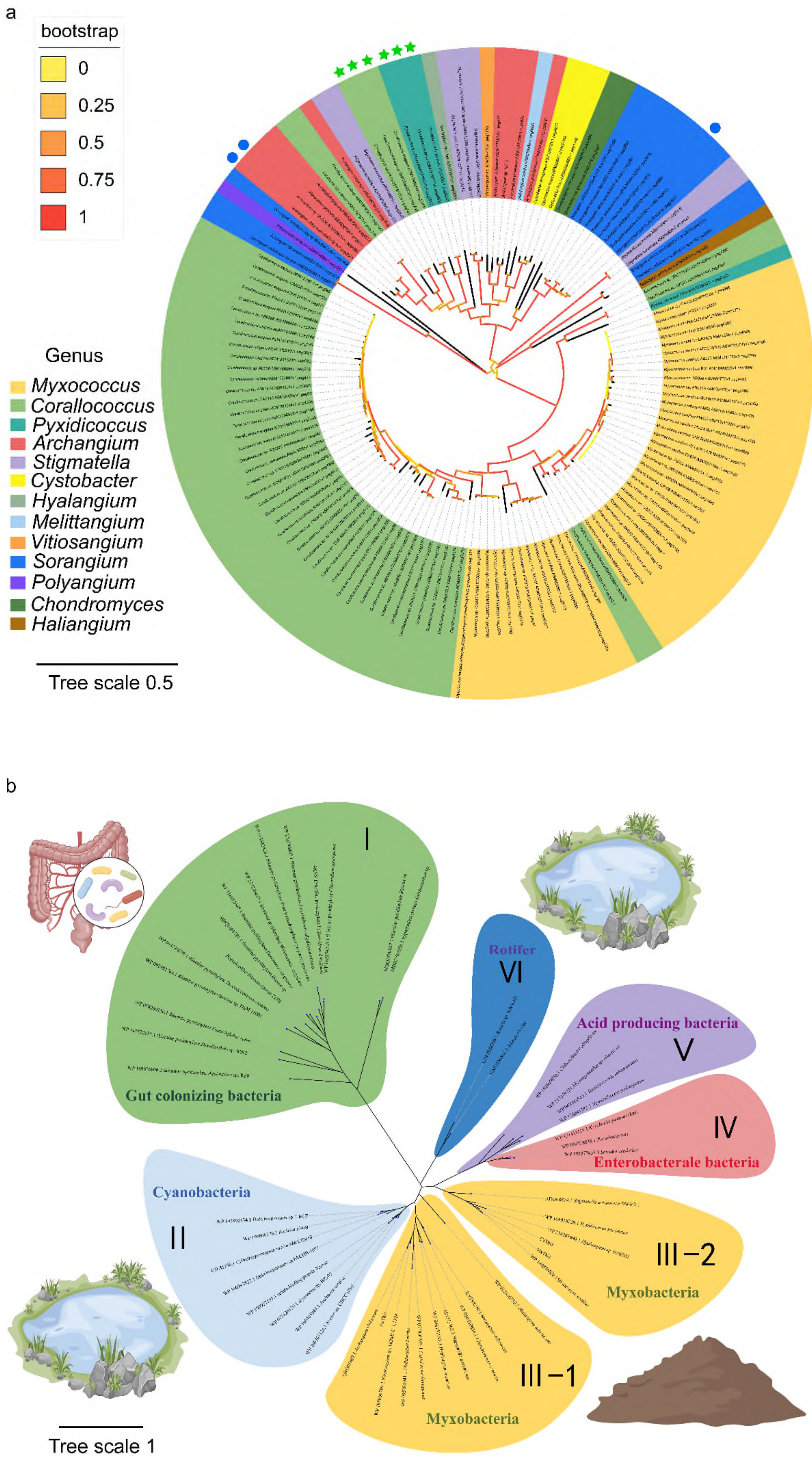

a
bootstrap
0
0.25
0.5
0.75
1
Genus
Myxococcus
Corallococcus
Pyxidicoccus
Archangium
Stigmatella
Cystobacter
Hyalangium
Melittangium
Vitiosangium
Sorangium
Polyangium
Chondromyces
Haliangium
Tree scale 0.5
b
I
Gut colonizing bacteria
Rotifer
VI
Acid producing bacteria
V
IV
Enterobacterale bacteria
Cyanobacteria
II
III−2
Myxobacteria
III−1
Myxobacteria
Tree scale 1

Supplementary Fig. 16 Distribution of CcThi1 homologs in myxobacterial taxa and identified thiaminases from different species in representative habitats. a, Phylogenetic tree analysis of CcThi1 homologs in myxobacterial taxa based on 138 sequences using the neighbor-joining method. Different colors represent different genera. A blue circle indicates no signal peptide, while a green pentagram indicates the presence of a non-lipoprotein signal peptide. **b,** Distribution of thiaminase I in the ecosystem. A phylogenetic tree was established based on 48 related sequences from selected species using the maximum likelihood method. The sequences were classified into 6 major categories (6 colors): Gut colonizing bacteria, Cyanobacteria, Myxobacteria, Enterobacteriales, Acid-producing bacteria and Rotifera. Cyanobacteria and rotifers are aquatic, gut colonizing bacteria are mainly isolated from fecal matter or animal intestines, while myxobacteria are mainly soil dwelling bacteria. The cartoon image was drawn by Figdraw.

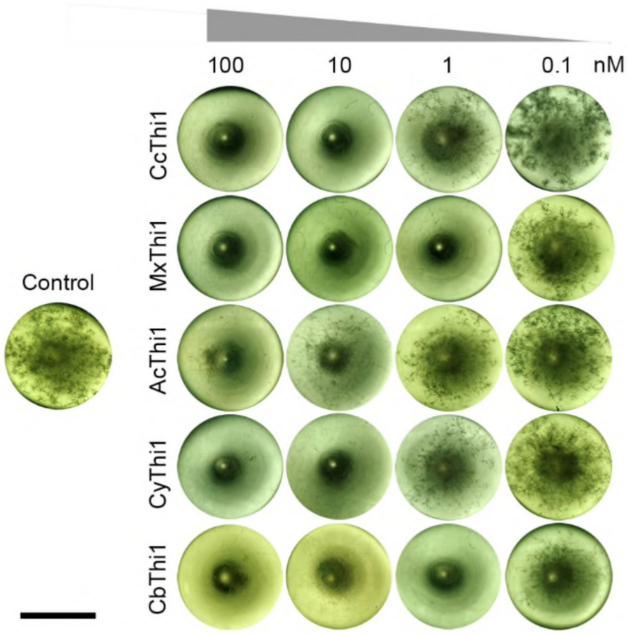

Supplementary Fig. 17 Effects of CcThi1 homologs from DK1622 (MxThi1), *Cystobacter* sp. 1404 (CyThi1) and *Archangium* sp. SDU8 (AcThi1) on the growth of P6497, compared to *Clostridium botulinum* CbThi1 as positive control. Agar discs were removed from the edge of an actively growing P6497 colony and transferred to the center of 96-well plates containing 90 µL of liquid 10% V8 medium and 10 µL of the indicated additives. The growth of P6497 was observed using a stereomicroscope (Nikon SMZ-10) after 20 h of incubation at 25 °C. Scale bar, 5 mm.

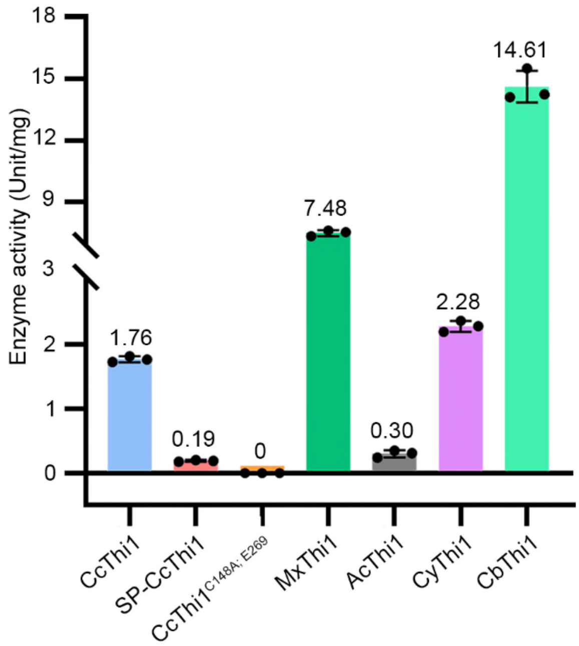

Supplementary Fig. 18 Activity determination of thiaminase CcThi1 homologs. The enzyme activity of CcThi1 homologs was measured using thiamine and 4-NTP as the substrate and nucleophile, respectively. CcThi1 from strain EGB; SP-CcThi1 from strain EGB with signal peptide; CcThi1$^{C148A;\ E269}$ was the CcThi1 variant with substitutions of conserved active-site residues C148 and E269; MxThi1 from strain DK1622; AcThi1 from *Archangium* sp. SDU8; CyThi1 from *Cystobacter* sp. 1404; CbThi1 from *Clostridium botulinum.* The data represent the means ± SEM (n = 3 biological independent replicates). Source data are provided as a Source Data file.

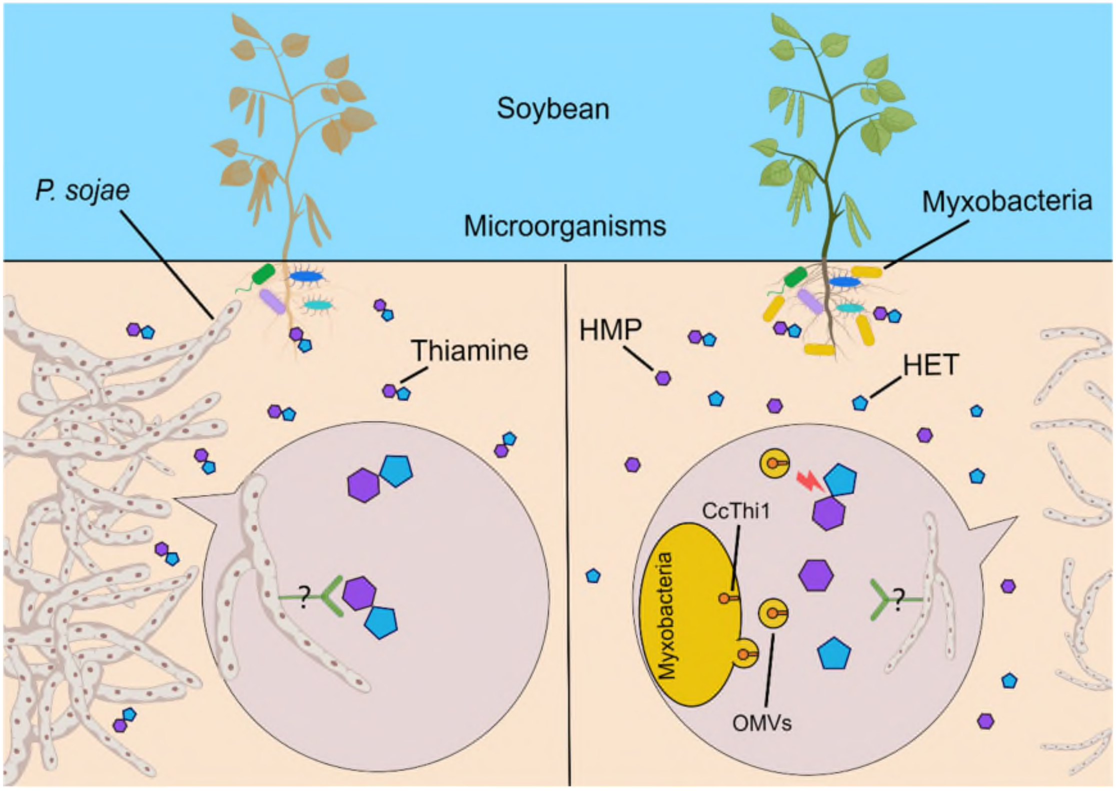

Supplementary Fig. 19 Suggested model of thiaminase CcThi1-mediated trophic interaction between myxobacteria and *Phytophthora*. We propose that myxobacteria remove thiamine produced by soybean plants as well as soil microorganisms through the secretion of thiaminase CcThi1 via OMVs, thus arresting the thiamine raiding behavior of *Phytophthora* by decomposing thiamine into HMP and HET, which failed to support the growth of *Phytophthora*. As members of the genus *Phytophthora* are thiamine auxotrophs and require exogenous sources of thiamine for growth, such as soybean root exudates and soil microorganisms, thiamine decomposition inhibits the growth of *Phytophthora* and controls soybean root rot disease by regulating the thiamine content in the soil environment. ? denotes an unknown thiamine transporter and the pattern diagram was drawn using Figdraw.

References

1. Xu S, *et al.* Fusarium fruiting body microbiome member *Pantoea agglomerans* inhibits fungal pathogenesis by targeting lipid rafts. *Nat. Microbiol.* **7**, 831-843 (2022).

2. OR RM. Mutants of *Escherichia coli*. *J. Bacteriol.* **60**, 17 (1950).

3. Liu X, *et al.* Arsenic Induced Phytate Exudation, and Promoted $FeAsO_4$ Dissolution and Plant Growth in As-Hyperaccumulator Pteris vittata. *Environ. Sci. Technol.* **50**, 9070-9077 (2016).

4. Samoylov VM, Tucker DR, Thibaud-Nissen F, Parrott WA. A liquid medium-based protocol for rapid regeneration from embryogenic soybean cultures. *Plant Cell Rep.* **18**, 49-54 (1998).

5. Chibucos MC, Morris PF. Levels of polyamines and kinetic characterization of their uptake in the soybean pathogen Phytophthora sojae. Appl Environ Microb 72, 3350-3356 (2006).

6. Han XS, *et al.* The Plant-Beneficial Rhizobacterium *Bacillus velezensis* FZB42 Controls the Soybean Pathogen *Phytophthora sojae* Due to Bacilysin Production. *Appl. Environ. Microbiol.* **87**, e01601-21 (2021).

7. Gao CY, *et al.* Pathogen manipulation of chloroplast function triggers a light-dependent immune recognition. *Proc. Natl. Acad. Sci. U. S. A.* **117**, 9613-9620 (2020).

8. Shi XQ, Wegener-Feldbrugge S, Huntley S, Hamann N, Hedderich R, Sogaard-Andersen L. Bioinformatics and experimental analysis of proteins of two-component systems in *Myxococcus xanthus*. *J. Bacteriol.* **190**, 613-624 (2008).

9. Alley W, Tao L, Shion H, Yu YQ, Rao CM, Chen WB. UPLC-MS assessment on the structural similarity of recombinant human erythropoietin (rhEPO) analogues from manufacturers in China for attribute monitoring. *Talanta* **220**, 121335 (2020).

10. Wu M, Papish ET, Begley TP. Mechanistic studies on thiaminase I: Identification of the product of thiamin degradation in the absence of the nucleophilic cosubstrate. *Bioorganic Chem.* **28**, 45-48 (2000).

11. Li Z, *et al.* A novel outer membrane β-1, 6-glucanase is deployed in the predation of fungi by myxobacteria. *ISME J.* **13**, 2223-2235 (2019).

12. Ma ZC, *et al.* A *Phytophthora sojae* Glycoside Hydrolase 12 Protein Is a Major Virulence Factor during Soybean Infection and Is Recognized as a PAMP. *Plant Cell* **27**, 2057-2072 (2015).

13. Bienapfl JC, Malvick DK, Percich JA. Specific molecular detection of *Phytophthora sojae* using conventional and real-time PCR. *Fungal Biol.* **115**, 733-740 (2011).

14. Kim D, Langmead B, Salzberg SL. HISAT: a fast spliced aligner with low memory requirements. *Nat.Methods* **12**, 357-360 (2015).

15. Trapnell C, Hendrickson DG, Sauvageau M, Goff L, Rinn JL, Pachter L. Differential analysis of gene regulation at transcript resolution with RNA-seq. *Nat. Biotechnol.* **31**, 46-53 (2013).

16. Hodgkin J, Kaiser D. Cell-to-cell stimulation of movement in nonmotile mutants of *Myxococcus*. *Proc. Natl. Acad. Sci. U. S. A.* **74**, 2938-2942 (1977).

17. Jensen NB, *et al.* EasyClone: method for iterative chromosomal integration of multiple genes in *Saccharomyces cerevisiae*. *FEMS Yeast Res.* **14**, 238-248 (2014).

18. Jiang X, *et al.* Impact of spatial organization on a novel auxotrophic interaction among soil microbes. *ISME J.* **12**, 1443-1456 (2018).
