# Supplementary material for: Myxobacteria restrain Phytophthora invasion by scavenging thiamine in soybean rhizosphere via outer membrane vesicle-secreted thiaminase I
Source: Nat Commun. 2023 Sep 13;14:5646. doi: 10.1038/s41467-023-41247-0 (PMC10499793; doi:10.1038/s41467-023-41247-0)
Supplement: Supplementary file 2 — Reporting Summary [file 41467_2023_41247_MOESM2_ESM.pdf]

nature portfolio

Corresponding author(s): Zhoukun Li, Zhongli Cui

Last updated by author(s): Aug 23, 2023

Reporting Summary

Nature Portfolio wishes to improve the reproducibility of the work that we publish. This form provides structure for consistency and transparency in reporting. For further information on Nature Portfolio policies, see our Editorial Policies and the Editorial Policy Checklist.

Statistics

For all statistical analyses, confirm that the following items are present in the figure legend, table legend, main text, or Methods section.

n/a	Confirmed	
☐	☒	The exact sample size (n) for each experimental group/condition, given as a discrete number and unit of measurement
☐	☒	A statement on whether measurements were taken from distinct samples or whether the same sample was measured repeatedly
☐	☒	The statistical test(s) used AND whether they are one- or two-sided<br>*Only common tests should be described solely by name; describe more complex techniques in the Methods section.*
☒	☐	A description of all covariates tested
☒	☐	A description of any assumptions or corrections, such as tests of normality and adjustment for multiple comparisons
☐	☒	A full description of the statistical parameters including central tendency (e.g. means) or other basic estimates (e.g. regression coefficient) AND variation (e.g. standard deviation) or associated estimates of uncertainty (e.g. confidence intervals)
☐	☒	For null hypothesis testing, the test statistic (e.g. F, t, r) with confidence intervals, effect sizes, degrees of freedom and P value noted<br>*Give P values as exact values whenever suitable.*
☒	☐	For Bayesian analysis, information on the choice of priors and Markov chain Monte Carlo settings
☒	☐	For hierarchical and complex designs, identification of the appropriate level for tests and full reporting of outcomes
☐	☒	Estimates of effect sizes (e.g. Cohen's d, Pearson's r), indicating how they were calculated

Our web collection on statistics for biologists contains articles on many of the points above.

Software and code

Policy information about availability of computer code

Data collection	Hitachi SU8010 for scanning electron microscope; RT-qPCR data were collected with ABI 7500 real-time PCR system; UPLC-MS analysis (G2-XS QTof, Waters); Illumina HiSeqTM 2500/4000 platform for RNA-seq; The membrane was visualized using an Odyssey (LI-COR) scanner;fluorescence detection was performed using confocal laser scanning microscopy (CLSM, LeicaTCSSP3, Germany);The growing P. sojae mycelia were observed using a stereomicroscope (Nikon SMZ-10, SMZ25);stained gel analyzed by NanoLC-ESI-MS/MS. MassLynx 4.1 was used for data collection and processing.
Data analysis	MEGA version 7.0.26; ClustalW version 2.0; SPSS (ver. 21.0; IBM Corp., Armonk, NY, USA); GraphPad Prism 8.0.2 (GraphPad Software Inc., USA);The reads were mapped to the P. sojae genome v1.1 (https://mycocosm.jgi.doe.gov/Physo1_1/Physo1_1.home.html) using HISAT2 with default parameters ;By using the Cuffdiff 2, the expression of each P. sojae gene was calculated by normalizing it to the fragment per kilobase of exon per million mapped reads (FPKM) value, and differentially expressed genes (DEGs) were identified.

For manuscripts utilizing custom algorithms or software that are central to the research but not yet described in published literature, software must be made available to editors and reviewers. We strongly encourage code deposition in a community repository (e.g. GitHub). See the Nature Portfolio guidelines for submitting code & software for further information.

Data

Policy information about availability of data

All manuscripts must include a data availability statement. This statement should provide the following information, where applicable:

- Accession codes, unique identifiers, or web links for publicly available datasets
- A description of any restrictions on data availability
- For clinical datasets or third party data, please ensure that the statement adheres to our policy

Raw sequencing data have been deposited at the NCBI SRA database (BioProject accession number: PRJNA909170). The nucleotide sequences of the ccthi1 gene of strain EGB (Genbank: WP_223633232.1), the mxthi1 gene of strain DK1622 (GenBank: ABF86831.1), the acthi1 gene of Archangium sp. SDU8 (GenBank: QRO01607.1) and the cythi1 gene of Cystobacter sp. 1404 (GenBank: OP978502) have been deposited in the NCBI nucleotide database. The data that support this study are available within the article and its Supplementary Information files. Source data are provided with this paper.

Research involving human participants, their data, or biological material

Policy information about studies with human participants or human data. See also policy information about sex, gender (identity/presentation), and sexual orientation and race, ethnicity and racism.

Reporting on sex and gender	Not applicable. Because no human research in this study
Reporting on race, ethnicity, or other socially relevant groupings	Not applicable. Because no human research in this study
Population characteristics	Not applicable. Because no human research in this study
Recruitment	Not applicable. Because no human research in this study
Ethics oversight	Not applicable. Because no human research in this study

Note that full information on the approval of the study protocol must also be provided in the manuscript.

Field-specific reporting

Please select the one below that is the best fit for your research. If you are not sure, read the appropriate sections before making your selection.

[x] Life sciences [ ] Behavioural & social sciences [ ] Ecological, evolutionary & environmental sciences

For a reference copy of the document with all sections, see nature.com/documents/nr-reporting-summary-flat.pdf

Life sciences study design

All studies must disclose on these points even when the disclosure is negative.

Sample size	No sample size calculation was performed. n=3 biological triplicates were performed.
Data exclusions	No data were excluded from the analyses.
Replication	Biochemical experiments were each replicated three times. All experiments performed in this study were successful.
Randomization	Sample allocation was random in our study.
Blinding	The investigators were blinded to group allocation during data collection and analysis in this study.

Reporting for specific materials, systems and methods

We require information from authors about some types of materials, experimental systems and methods used in many studies. Here, indicate whether each material, system or method listed is relevant to your study. If you are not sure if a list item applies to your research, read the appropriate section before selecting a response.

Materials & experimental systems

n/a	Involved in the study
☐	☒ Antibodies
☒	☐ Eukaryotic cell lines
☒	☐ Palaeontology and archaeology
☒	☐ Animals and other organisms
☒	☐ Clinical data
☒	☐ Dual use research of concern
☐	☒ Plants

Methods

n/a	Involved in the study
☒	☐ ChIP-seq
☒	☐ Flow cytometry
☒	☐ MRI-based neuroimaging

Antibodies

Antibodies used	The peptide sequence CGGRPYPNQRFPENR derived from CcThi1 was synthesized for the production of anti-CcThi1 antibodies by immunizing rabbits (C892NGD210-1, GenScript, China) , the peptide of CcThi1 shares 76.66% sequence identity with MxThi1 of DK1622. Anti-rabbit IRDye 800CW antibody (Odyssey, catalog no. 926–32211) .
Validation	Anti-CcThi1 antibodies was validated by ELISA experiments. Indirect ELISA<br>Coating Antigen(s): Free Peptide<br>Coating Concentration: 4 μg/ml, 100 μl/well<br>Coating Buffer: Phosphate Buffered Saline, pH 7.4<br>Secondary Antibody: Anti-Rabbit IgG Fc Monoclonal Secondary Antibody (Min X Hu, Ms, Rt, Sh, Bv, Gt, Camel) (HRP conjugate) (GenScript, Cat. No. A01856)<br>Anti-rabbit IRDye 800CW antibody: https://www.licor.com/bio/reagents/irdye-800cw-goat-anti-rabbit-igg-secondary-antibody.

Dual use research of concern

Policy information about dual use research of concern

Hazards

Could the accidental, deliberate or reckless misuse of agents or technologies generated in the work, or the application of information presented in the manuscript, pose a threat to:

No	Yes
☒	☐ Public health
☒	☐ National security
☒	☐ Crops and/or livestock
☒	☐ Ecosystems
☒	☐ Any other significant area

Experiments of concern

Does the work involve any of these experiments of concern:

No	Yes
☒	☐ Demonstrate how to render a vaccine ineffective
☒	☐ Confer resistance to therapeutically useful antibiotics or antiviral agents
☒	☐ Enhance the virulence of a pathogen or render a nonpathogen virulent
☒	☐ Increase transmissibility of a pathogen
☒	☐ Alter the host range of a pathogen
☒	☐ Enable evasion of diagnostic/detection modalities
☒	☐ Enable the weaponization of a biological agent or toxin
☒	☐ Any other potentially harmful combination of experiments and agents
